# Incidence of *Cyp51 A* Key Mutations in *Aspergillus fumigatus*—A Study on Primary Clinical Samples of Immunocompromised Patients in the Period of 1995–2013

**DOI:** 10.1371/journal.pone.0103113

**Published:** 2014-07-29

**Authors:** Birgit Spiess, Patricia Postina, Mark Reinwald, Oliver A. Cornely, Axel Hamprecht, Martin Hoenigl, Cornelia Lass-Flörl, Peter-Michael Rath, Jörg Steinmann, Thomas Miethke, Melchior Lauten, Silke Will, Natalia Merker, Wolf-Karsten Hofmann, Dieter Buchheidt

**Affiliations:** 1 Department of Hematology and Oncology, University Hospital Mannheim, University of Heidelberg, Mannheim, Germany; 2 Department I of Internal Medicine, University Hospital of Cologne, Cologne; Clinical Trials Centre Cologne, ZKS Köln, BMBF 01KN1106, and Centre for Integrated Oncology CIO Köln Bonn, and Cologne Excellence Cluster on Cellular Stress Response in Aging-Associated Diseases (CECAD), University of Cologne, Cologne, Germany; 3 Institute for Medical Microbiology, Immunology and Hygiene, University Hospital of Cologne, Cologne, Germany; 4 Section of Infectious Diseases and Tropical Medicine & Department of Pulmonology, University Hospital of Internal Medicine, Graz Medical University, Graz, Austria; 5 Section of Hygiene and Medical Microbiology, Medical University of Innsbruck, Innsbruck, Austria; 6 Institute of Medical Microbiology, University Hospital of Essen, Essen, Germany; 7 Institute of Medical Microbiology and Hygiene, University Hospital Mannheim, Mannheim, Germany; 8 Pediatric Hematology and Oncology, University Hospital Schleswig-Holstein, Campus Lübeck, Lübeck, Germany; Leibniz Institute for Natural Products Research and Infection Biology- Hans Knoell Institute, Germany

## Abstract

As the incidence of azole resistance in *Aspergillus fumigatus* is rising and the diagnosis of invasive aspergillosis (IA) in immunocompromised patients is rarely based on positive culture yield, we screened our *Aspergillus* DNA sample collection for the occurrence of azole resistance mediating *cyp51 A* key mutations. Using two established, a modified and a novel polymerase chain reaction (PCR) assays followed by DNA sequence analysis to detect the most frequent mutations in the *A. fumigatus cyp51 A* gene conferring azole resistance (TR34 (tandem repeat), L98H and M220 alterations). We analyzed two itraconazole and voriconazole and two multi-azole resistant clinical isolates and screened 181 DNA aliquots derived from clinical samples (blood, bronchoalveolar lavage (BAL), biopsies, cerebrospinal fluid (CSF)) of 155 immunocompromised patients of our *Aspergillus* DNA sample collection, previously tested positive for *Aspergillus* DNA and collected between 1995 and 2013. Using a novel PCR assay for the detection of the *cyp51 A* 46 bp tandem repeat (TR46) directly from clinical samples, we found the alteration in a TR46/Y121F/T289A positive clinical isolate. Fifty stored DNA aliquots from clinical samples were TR46 negative. DNA sequence analysis revealed a single L98H mutation in 2010, two times the L98H alteration combined with TR34 in 2011 and 2012 and a so far unknown N90K mutation in 1998. In addition, four clinical isolates were tested positive for the TR34/L98H combination in the year 2012. We consider our assay of epidemiological relevance to detect *A. fumigatus* azole resistance in culture-negative clinical samples of immunocompromised patients; a prospective study is ongoing.

## Introduction

*Aspergillus* species cause invasive aspergillosis (IA), a life-threatening infection in immunocompromised patients. As triazole antifungal drugs (itraconazole, voriconazole, posaconazole) represent prophylaxis or standard first-line therapy against IA, emerging of resistance is of clinical concern. Infections due to azole resistant *Aspergillus fumigatus* (*A. fumigatus*) have increased over recent years [1]–[3], and are associated with markedly higher case fatality rates in particular among patients with azole resistant IA [4]. To date target-site modification is the main observed mechanism conferring azole drug resistance. The most important target, the 14α-sterol demethylase, is encoded by the *cyp51 A* gene of *A. fumigatus*. It has been reported that mutational hotspots and/or increased *cyp51 A* expression mediated by tandem repeats (TR34/TR46) promoter alterations in this gene cause azole drug resistance and, from a clinical point of view, fatal treatment failure.

The first TR34/L98H positive isolate was found in the Netherlands in 1998 [5]. The first hematological patient suffering from a multi-azole resistant IA was detected in Spain in 2003 and described in 2013 [6]. In 2007 Verweij et al., described recent clinical isolates of two hematological patients with multi-azole *A. fumigatus* infections who died during azole therapy [7]. Van der Linden et al. evaluated *A. fumigatus* isolates collected from 2007 to 2009 and from 2009 to 2011 and published in 2011 eight well documented hematological patients suffering from multi-azole resistant IA caused by *cyp51 A* TR34/L98H alterations. Seven of these patients died [4]. In 2013 the same group described three additional hematological patients with multi-azole resistant *A. fumigatus* infections showing a new combination of *cyp51 A* alterations (TR46/Y212F/T289A) [8]. In France only one hematological patient with azole resistant IA and the TR34/L98H alteration of the *cyp51 A* gene was detected [9]. In Germany, the first hematological patient with multi-azole resistant IA was published in 2012 [10]. Recently, Bader et. al. described 17 azole resistant clinical *A. fumigatus* isolates from a total of 527 patients investigated in Germany; of these isolates only one was originated from a hematological patient [11].

The diagnosis of azole resistance is mainly based on the positive culture of an isolate from a clinical specimen, susceptibility testing, and culture-based PCR assays [12]–[14]. At least in hematological patients at high risk for IA evident *Aspergillus* culture yields from the sites of infection are scarce and a culture-based diagnosis of IA is rarely achieved [15], [16].

To overcome these diagnostic limitations we established PCR assays to detect three key azole resistance mutations of *A. fumigatus cyp51 A* gene directly in primary clinical samples from patients with hematological malignancies [17]. Up to now only one other PCR assay has been described, to detect *cyp51 A* gene mutations investigating directly clinical samples, not isolates. That assay detects the mutations directly in sputum samples from patients with chronic pulmonary diseases; however, has failed to yield results due to “insufficient sample remaining” in samples from a few patients suffering from invasive pulmonary aspergillosis (IPA) [18].

Our group described the non-culture-based detection of *A. fumigatus cyp51 A* mutations directly in clinical samples of three hematological patients with IA [17]. In our above mentioned study [17] one of these three patients was described and confirmed microbiologically as the first hematological patient in Germany infected with a multi-azole resistant *A. fumigatus* strain carrying the TR34/L98H *cyp51 A* alterations [10].

In the present study we used our combined molecular approach to detect *Aspergillus* genome directly in clinical samples (first step) followed by the analysis of *cyp51 A* linked resistance mediating key mutations (second step). We screened 181 clinical samples (blood, bronchoalveolar lavage (BAL), tissue biopsies, cerebrospinal fluid (CSF)) of 155 immunocompromised patients included in our *Aspergillus* DNA sample compilation collected between 1995 and 2013.

## Materials and Methods

### Patients

For the determination of mutations in the *A. fumigatus cyp51 A* gene conferring azole resistance (TR34/TR46, L98H and M220 alterations), we investigated clinical samples (blood, BAL, tissue biopsies, CSF) of 155 immunocompromised patients mainly with hematological malignancies, previously tested positive for *Aspergillus* DNA and collected between 1995 and 2013. In the retrospective analysis the number of samples ranged from 1 to 19 per year. Underlying diseases of the patients are shown in Table 1.

**Table 1:** Primary diseases of 155 investigated immunocompromised patients using the azole resistance PCR assays.

Disease	Number of patients	Proven IA	Probable IA
ALL	16	4	2
AML	42	4	2
Aplastic anemia	2		1
Arteritis temporalis	1		
Autoimmune neutropenia	1	1	
CLL	10		
CML	3		
Combined immunodeficiency	1		1
HIV	4		
Hodgkin's disease	2	1	
MDS	7		1
Myeloma	1		
NHL	28	5	
OMF	1		
Solid tumor	7	1	
Other hematological malignancies with suspicious lung infiltrates	21	1	
Other hematological malignancies with immunosuppression	1		
Other hematological malignancies not specified	7		

AML, acute myeloid leukemia; ALL, acute lymphatic leukemia; CLL, chronic lymphatic leukemia; CML, chronic myeloid leukemia; CMML, chronic myelomonocytic leukemia; COPD, chronic obstructive pulmonary disease; HIV, human immunodeficiency virus; JMML, juvenile myelomonocytic leukemia, MDS, myelodysplastic syndrome; NHL, non Hodgkin lymphoma; OMF, osteomyelofibrosis.

Samples submitted to the scientific laboratory of the Department of Hematology and Oncology of the Mannheim University Hospital, Germany, for diagnosing IA between July of 1995 and October 2013, were analyzed to elucidate PCR performance in these samples. Immunocompromised patients at high risk for fungal infections were included in this retrospective analysis. Patients' data had been pseudonymized previously. Written informed consent of patients or legal representatives had been acquired prior to sampling and analysis was done according to Good Clinical Practice (GCP) guidelines as well as in concordance with the Declaration of Helsinki. The study was approved by the local Ethics Committee (Ethics Committee of the Faculty of Medicine Mannheim, University of Heidelberg, Germany; reference number 2011-280N-MA).

### Clinical samples

Blood samples were obtained by venipuncture under sterile conditions and placed in a sterile vessel containing potassium EDTA to a final concentration of 1.6 mg EDTA per ml of blood. The sample volume was 5 to 7 ml.

Bronchoscopy and bronchoalveolar lavage (BAL) was performed according to standardized operating procedures as described elsewhere [19], and BAL samples were obtained in a sterile vessel without conservation media. The mean sample volume was 10 ml. For analysis the BAL sample was processed. Tissue samples were obtained by needle biopsies (lung, liver, kidney) or surgical procedures (brain, other samples) under sterile conditions.

### Strains and growth conditions

*A. fumigatus* wildtype strain (DSM 819) was obtained from the Deutsche Sammlung von Mikroorganismen und Zellkulturen GmbH, Braunschweig, Germany, and the Institute of Medical Microbiology and Hygiene, Mannheim University Hospital, Mannheim, Germany.

Two itraconazole and voriconazole resistant *A. fumigatus* clinical isolates were obtained from the University Hospitals of Cologne and the University Hospital of Essen. Strain A 4813 showed the following MIC (minimum inhibitory concentration) values obtained by EUCAST reference microdilution method: voriconazole 2.0, itraconazole >16.0, posaconazole 0.5 mg/L [10]. Strain vv3799 showed the following EUCAST MIC values: voriconazole 4.0, itraconazole ≥16.0, posaconazole 0.5 mg/L [20]. MICs were interpreted according to EUCAST breakpoints [21]. Characterization of the multi-azole resistant clinical isolate (A12519) revealed the following E-test MIC values: itraconazole 8 mg/L, voriconazole >32 mg/L, posaconazole 0.75 mg/L, amphotericin B 0.38 mg/L.

The resistance patterns of the isolates A 68 and A 71 (Medical University of Innsbruck) revealed the following E-test MIC values: A 68: itraconazole >32 mg/L, voriconazole 2–4 mg/L, posaconazole 1.5–3 mg/L; A 71: itraconazole >32 mg/L, voriconazole 0.5–0.75 mg/L, posaconazole 1.5–3 mg/L.

Prior to DNA extraction fungal cultures were grown in BBL Malt Agar (Becton Dickinson/Heidelberg/Germany) for 72 h at 30°C.

### DNA extraction

DNA extraction from fungal cultures as well as from blood and BAL samples was performed using the phenol/chloroform extraction method as previously described [22], [23]. Tissue samples were processed additionally in liquid nitrogen for disruption. The tissue was sheared using a sterile scalpel in a sterile Petri dish under sterile conditions. The generated nuggets were transferred into a tissueTUBE used for processing of the sample in a cryoPREP workflow (Covaris; USA). The tissueTUBE was incubated in liquid nitrogen for 30–45 s until the sample was completely frozen. After freezing the tube was fitted into the cryoPREP workflow, were the tissue was pestled. The frozen tissue crumbs were transferred into a sterile 50 ml reaction tube and mixed with 1.5 ml 1 × PBS puffer. The tissue/PBS mixture was transferred into a 1.5 ml reaction tube and centrifuged at 13000 rmp for 10 min. Supernatant was discarded and the pellet was resuspendet in 250 µl 1 × PBS puffer.

### Primers for PCR assays

For amplification of the TR34 and M220 alterations, primer pairs were used as previously described [17] to perform a nested and a one step PCR assay. The amplification of the L98H alteration was performed using the primer combination Asp-L98H-F/Asp-L98H-R described by van der Linden et al., 2010 [24]. To predict the potential cross reactivities of the *A. fumigatus cyp51 A* primer sequences of Asp-L98H-F/Asp-L98H-R with human genomic DNA sequences, additional database alignments were performed by using the NCBI primer-BLAST service (http://www.ncbi.nlm.nih.gov/). The *A. fumigatus cyp51 A* gene specific TR46 primer pairs were designed from the promoter region of the *A. fumigatus cyp51 A* DNA sequence (AF 338659.1) available in the GenBank database (http://www.ncbi.nlm.nih.gov/). To predict the potential cross reactivities of the *A. fumigatus cyp51 A* primer sequences with human genomic DNA sequences, additional database searches were performed by using the primer-BLAST service. The melting temperatures (T_m_s) of the primers and possible secondary structures were calculated using also the NCBI primer designing tool primer-BLAST (http://www.ncbi.nlm.nih.gov/). The synthetic oligonucleotides were synthesized by Eurofins MWG Operon/Ebersberg/Germany and diluted to 100 µM in ddH_2_O.

### PCR assays, specificity and sensitivity

**Figure 1:**
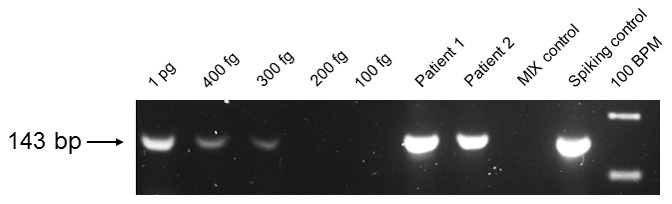
Determination of the improved one-step L98H PCR assay sensitivity. The determination was performed by Gelstar (Bio-Rad GmbH, Munich, Germany) stained agarose gel electrophoresis using serially diluted *A. fumigatus* wildtype DNA. The PCR amplicons of the patients were used for DNA sequencing analysis after elution from the agarose gel. BPM = base pair marker; Spiking control = 100 ng human DNA +50 ng *A. fumigatus* wildtype DNA.

PCR conditions for the TR34 and M220 PCR assays have been described previously [17]. The novel L98H PCR assay using the primer pair Asp-L98H-F and Asp-L98H-R published by van der Linden [24] was performed as one-step PCR assay. The generated PCR fragment was 143 bp in length. Following PCR conditions were used: total volume, 25 µl; 3 µl template DNA (approximately 100 ng human DNA plus an unknown amount of *A. fumigatus* DNA), 3 mM MgCl_2_, 0.25 mM each deoxynucleoside triphosphate, 1 U of *Taq* polymerase (Invitrogen GmbH, Karlsruhe, Germany), 20 pmol of each primer; DNA thermal cycler, 5 min of initial denaturation at 94°C, 40 cycles of 94°C for 45 s, 52°C for 1 min, and 72°C for 1 min, and final extension at 72°C for 10 min. Sensitivity of the optimized L98H PCR assays was determined using serially diluted *A. fumigatus* wildtype DNA as template (Figure 1).

The TR46 PCR assay was performed as nested PCR assay. For the first PCR the primer pair TR46long-S (5′-AAGCACTCTGAATAATTTACA) and TR46long-AS (5′-ACCAATATAGGTTCATAGGT-3′) was used for amplification of a 240 bp DNA fragment. In the second PCR step a 103 bp DNA fragment is generated using the primer pair TR46short-S (5′-GAGTGAATAATCGCAGCACC-3′) and TR46short-AS (5′-CTGGAACTACACCTTAGTAATT). Sensitivity of the TR46 PCR assay was determined using serially diluted *A. fumigatus* wildtype DNA as template. PCRs were performed under the following conditions: first step: total volume, 25 µl; 3 µl template DNA (approximately 100 ng human DNA plus an unknown amount of *A. fumigatus* DNA), 3 mM MgCl_2_, 0.25 mM each deoxynucleoside triphosphate, 1 U of *Taq* polymerase (Invitrogen GmbH, Karlsruhe, Germany), 20 pmol of each primer; DNA thermal cycler, 2 min of initial denaturation at 94°C, 23 cycles of 94°C for 45 s, 52°C for 1 min, and 72°C for 1 min, and final extension at 72°C for 5 min. Second step: total volume 25 µl: template: 3 µl of the first step PCR mixture, other components are on par with the first step mixture, DNA thermal cycler, 2 min of initial denaturation at 94°C, 35 cycles of 94°C for 45 s, 56°C for 1 min, and 72°C for 1 min, and final extension at 72°C for 5 min.

To test cross reactivity of the designed primer pairs with human genomic DNA, we investigated a sample adopted in the PCR assays containing a mixture of 100 ng human genomic DNA and 50 ng of *A. fumigatus* wildtype DNA. PCR products were analyzed by agarose gel analysis stained with GelStar (Bio-Rad GmbH, Munich, Germany).

### Control

The established nested PCR assay for the detection of the 46 bp tandem repeat (TR46) directly from clinical samples (BAL, tissue biopsies) as a marker of the TR46/Y121F/T289A genotype was tested using DNA of a TR46/Y121F/T289A positive, multi-azole resistant clinical isolate (A12519).

### Sequence analysis

The PCR products were used for mandatory sequence analysis. The amplicons were purified using the MiniElute PCR purification kit (Qiagen, Hilden, Germany) and a minimum of 50 ng DNA was sequenced (Sequiserve, Vaterstetten, Germany). To detect potential mutations in the PCR products analyzed by DNA sequence analysis, the sequence of the products was compared to the sequence of the *A. fumigatus cyp51 A* wildtype sequence using the NCBI alignment service AlignSequenceNucleotideBlast (http://www.ncbi.nlm.nih.gov/).

## Results

**Figure 2:**
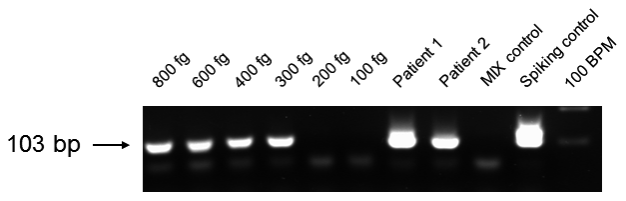
Determination of the TR46 nested PCR assay sensitivity. The determination was performed by Gelstar (Bio-Rad GmbH, Munich, Germany) stained agarose gel electrophoresis using serially diluted *A. fumigatus* wildtype DNA. The PCR amplicons of the patients were used for DNA sequencing analysis after elution from the agarose gel. BPM = base pair marker; Spiking control = 100 ng human DNA +50 ng *A. fumigatus* wildtype DNA.

Two established [17], one improved and a novel PCR assay were used for the detection of the most common azole resistance mediating alterations in the *A. fumigatus cyp51 A* gene directly from clinical samples stored in our *Aspergillus* DNA collection. The assays showed different detection thresholds for genomic *A. fumigatus* DNA. The sensitivity of the established PCR assays was 600 fg of *A. fumigatus* DNA for the TR34 mutation and 4 pg for the M220 mutation. For the improved L98H PCR assay, the determined sensitivity was 300 fg (Figure 1) in comparison to the earlier published L98H PCR with a sensitivity of 6 pg [17] and for the TR46 PCR assay 300 fg of *A. fumigatus* DNA (Figure 2).

Using both, the improved and the novel PCR assay, when investigating DNA of two patients' samples (lung biopsy of patient 1 with acute myeloid leukemia (AML); brain tissue biopsy of patient 2 suffering from T-lymphoblastic acute leukemia (T-ALL)) resulted in positive signals, exemplarily as a proof of principle (Figure 1 and 2). DNA sequence analysis of the *A. fumigatus* wildtype PCR amplicons confirmed the wildtype sequence of the *cyp51 A* gene, showing that *A. fumigatus cyp51 A* DNA fragments were amplified by the use of the particular PCR assays. DNA sequence analysis is mandatory to detect the mutations in the amplified *cyp51 A* gene fragments.

Stored samples (n = 181) of 155 immunocompromised patients (Table 1), positive in our nested diagnostic *Aspergillus* PCR assay [23], were analyzed with the L98H, M220 and TR34 PCR assays and consecutive DNA sequence analysis both to confirm the feasibility of our method and for epidemiological evaluation of the occurrence of the most common azole resistance mediating mutations in the *A. fumigatus cyp51 A* gene in immunosuppressed patients in Germany. Patients' characteristics are shown in Table 1.

Using the most sensitive L98H PCR assay, 106 from 181 investigated samples were negative. Twenty-six samples were positive in the L98H PCR assay alone, but revealed no mutations in the sequence analysis. Seventeen samples were positive using the L98H and TR34 PCR assays, but they also showed no mutations. Thirty-two samples were positive in all three PCR assays, one sample revealed a L98H mutation alone and two samples showed the L98H mutation in combination with the TR in the sequence analysis (Table 2).

**Table 2:** Azole resistance PCR results of 181 investigated clinical samples.

Number of clinical samples	L98H PCR	L98H alteration	TR34 PCR	TR34 alteration	M220 PCR	M220 alteration
**106**	**-**					
**26**	**+**	**-**	**-**			
**17**	**+**	**-**	**+**	**-**	**-**	
**32**	**+**	**3**	**+**	**2**	**+**	**0**

106 samples including all blood samples were PCR negative in the most sensitive L98H PCR assay. Investigation of 32 samples revealed positive PCR results in all three PCR assays and showed three times the L98H mutation, two times in combination with the TR34.

These 181 clinical samples encompassed 25 blood, 120 BAL, 17 tissue biopsies and 19 CSF samples. All blood samples were PCR-negative, 59 BAL samples resulted in positive L98H PCR signals whereas one of the BAL samples revealed the L98H mutation in combination with the TR34 alteration in the sequence analysis. Nine tissue biopsies were L98H PCR positive, revealing the L98H mutation in a lung biopsy and the L98H/TR combination in a brain biopsy. Seven CSF samples were L98H PCR positive but showed no mutations (Table 3).

**Table 3:** Azole resistance PCR results of 25 investigated blood (PB = peripheral blood), 120 BAL, 17 tissue biopsies and 19 CSF samples.

Clinical sample type	n	L98H PCR positive	L98H alter-ation	TR34 PCR positive	TR34 alter-ation	M220 PCR positive	M220 alter-ation
**PB**	**25**	**0**	**0**	**0**	**0**	**0**	**0**
**BAL**	**120**	**59**	**1**	**39**	**1**	**25**	**0**
**Tissue biopsies**	**17**	**9**	**2**	**7**	**1**	**5**	**0**
**CSF**	**19**	**7**	**0**	**3**	**0**	**2**	**0**

106 samples including all blood samples were PCR negative in the most sensitive L98H PCR assay. Investigation of 32 samples revealed positive PCR results in all three PCR assays and showed three times the L98H mutation, two times in combination with the TR34. n, number of clinical samples.

One clinical sample showing the L98H *cyp51 A* alteration alone was obtained from a patient with a preexisting chronic obstructive pulmonary disease (COPD) and AML with respiratory failure due to refractory lung infiltrates. A tissue biopsy of an unclear brain abscess of patient 2 suffering from acute T-lymphoblastic leukemia (T-ALL) was TR34/L98H positive, as described by our group in 2012 [17]. Another TR34/L98H positive sample was a BAL sample of a neutropenic AML patient (patient 3); in this case the infection was diagnosed first by a positive diagnostic nested *Aspergillus* PCR assay [23] in the BAL sample, followed by the molecular characterization of *cyp51 A* alterations using our azole resistance PCR assays with subsequent DNA sequence analysis revealing the TR34/L98H alterations. After the subsequent culture based confirmation of multiple azole resistance, the treatment was switched to liposomal amphotericin B. The patient recovered and is in complete remission from AML [10].

The results for these clinical samples in terms of *cyp51 A* alterations are summarized in Table 4.

**Table 4:** Characteristics of patients 1–3 and the corresponding clinical samples showing *cyp51 A* TR34/L98H alterations.

Patients	Sample	Diagnosis	Diagnostic significance	nested PCR result *Aspergillus*	*cyp51 A* TR34 alteration	*cyp51 A* L98H alteration	*cyp51 A* M220 alteration
**1**	**Lung biopsy**	**AML/COPD**	**probable**	**+**	**-**	**+**	**-**
**2**	**Brain abscess**	**T-ALL**	**probable**	**+**	**+**	**+**	**-**
**3**	**BAL**	**AML (lung infiltrate)**	**proven**	**+**	**+**	**+**	**-**

All clinical samples have been tested positive for *Aspergillus* DNA in our diagnostic nested PCR assay. Pts., patients; AML, acute myeloid leukemia; T-ALL, T-lymphoblastic acute leukemia; COPD, chronic obstructive pulmonary disease.

The epidemiological evaluation encompassing *Aspergillus* DNA aliquots positive in the diagnostic nested PCR assay between 1995 and 2013 revealed the first occurrence of a L98H mutation in the lung biopsy of the AML/COPD patient (patient 1/probable IA) in the year 2010, the L98H alteration in combination with the TR34 in the brain tissue sample of patient 2 (probable IA) with ALL in 2011 and in the BAL sample of patient 3 (proven IA) with AML in 2012. In summary in our series the incidence of these resistance mediating mutations investigating clinical samples directly amounts to 1.94% (3/155 patients) in a population of patients with hematological malignancies. Furthermore we investigated 50 clinical samples out of these 181 samples described above for the occurrence of TR46 alterations. DNA sequence analysis revealed no TR46 alterations in these series. The TR46 PCR assay was established recently, so that the investigation of clinical samples is still in progress.

Investigating *A. fumigatus* DNA of four isolates from 2012 for the occurrence of *cyp51 A* key mutations using our established PCR assays we detected the TR34/L98H alterations in the isolate vv3799 (Table 5). This case was reported elsewhere [20].

**Table 5:** Results of azole PCR based investigation of a multi-azole resistant *Aspergillus fumigatus* clinical isolate of a patient with heroin addict and aspergilloma, the corresponding clinical isolate of patient 3 suffering from AML and isolates A68 and A71 from immunocompromised patients.

A. fumigatus isolate	Resistance	Diagnosis	cyp51 A TR34 alteration	cyp51 A L98H alteration	cyp51 A M220 alteration
**vv3799**	**Itraconazole, voriconazole**	**Heroin addict, aspergilloma**	**+**	**+**	**-**
**A 4813 from BAL (pat. 3)**	**Itraconazole, voriconazole**	**AML, lung infiltrates**	**+**	**+**	**-**
**A 68**	**Itraconazole, voriconazole, posaconazole**	**Immuno-suppression**	**+**	**+**	**-**
**A 71**	**Itraconazole, voriconazole, posaconazole**	**Immuno-suppression**	**+**	**+**	**-**

PCR and DNA sequence analysis of all isolates revealed the TR34/L98H alterations.

The second itraconazole and voriconazole resistant isolate A 4813 was the corresponding isolate of patient 3, showing positive resistance PCR results and the TR34/L98H alterations in the BAL sample as mentioned in Table 4. The TR34/L98H alterations were also detected in the clinical isolate of this patient (Table 5), published as the first case with acute leukemia revealing the TR34/L98H alterations in Germany [10]. As a proof of principle, two multi-azole resistant isolates (A68, A71) originating from immunocompromised patients showed the TR34/L98H alterations in PCR and sequence analysis.

Investigating an *Aspergillus* PCR-positive BAL sample of a hematological patient suffering from chronic lymphatic leukemia (CLL) from 1998 we detected a N90K mutation next to the L98H mutation. The patient had received antifungal treatment with deoxycholate amphotericin B. There is no microbiological data of this patient available, so the influence or potential contribution on azole resistance of this novel mutation cannot be clarified.

## Discussion

In order to investigate the epidemiology of azole resistance, 181 clinical samples of 155 immunosuppressed patients were investigated for *A. fumigatus cyp51 A* mutations by PCR using two established and an improved PCR assay. Out of these samples fifty DNA aliquots were additionally tested with a novel PCR assay for the occurrence of the TR46 alteration as a marker of the TR46/Y121F/T289A genotype. Previously [17] we showed that the molecular azole resistance detection directly from primary clinical samples is feasible and of clinical benefit, because evident *Aspergillus* culture yields of blood, BAL or tissue samples are scarce and a culture-based diagnosis of IA is rarely achieved in patients with hematological malignancies -at high risk for IA-. Previous studies have shown repeatedly, that the fungal load is higher in samples originated directly from the site of infection than e.g. in blood [25]–[27]. Since the amount of *A. fumigatus* DNA is crucial when investigating directly primary clinical samples with azole resistance PCR assays, BAL, CSF and tissue samples are therefore most applicable for this method. This is true in particular for hematological patients with IA undergoing intensive antifungal therapy. However, it is not possible to amplify the whole *cyp51 A* gene due to the very low amount of high-molecular, intact fungal DNA in these clinical samples, so that we focused on the detection of four key mutations with single PCR assays.

Other PCR assays for the detection of *A. fumigatus cyp51 A* alterations have been described, however these assays were established to investigate fungal DNA solely from cultures derived from clinical isolates [12]–[14], [28], [29]. In addition to our azole resistance PCR assays published in 2012 [17] there is only one PCR-based assay described detecting *cyp51 A* mutations in clinical samples (sputum) from patients with underlying pulmonary diseases. Only a few patients in this study suffered from invasive pulmonary aspergillosis (IPA), however, these samples were not tested “due to insufficient sample remaining” [18].

According to recent studies, azole resistance in *Aspergillus fumigatus* isolates is primarily due to the TR34/L98H mutation and would thus be detected by our assays [4], [11], [30]–[33]. Howard et al. described more mutation variety in the Manchester patient population, so that our assay might work probably less well diagnosing azole resistance in this group of patients (mostly patients with underlying pulmonary diseases) [34]. Furthermore van der Linden et al. observed a new *cyp51 A*-mediated resistance mechanism (TR46/Y121F/T289A) in 21 azole-resistant isolates from 15 patients in six hospitals in the Netherlands. These TR46/Y121F/T289A isolates showed very high MICs to voriconazole (MIC≥16 mg/l) [8]. To cover this issue, we established an additional PCR assay to detect TR46 alteration as a marker of the TR46/Y121F/T289A genotype. The same resistance mutation combination has been described also in cystic fibrosis patients, recently [35]. In general, the development of azole resistance in *Aspergillus fumigatus* in cystic fibrosis patients due to TR34/L98H and other *cyp51 A* mutations has been published [36]–[38]. However, this group of patients is different to our severely immunocompromised hematological patients' population, since culture of fungal diagnostic from respiratory samples is mostly positive in cystic fibrosis patients; the amount of DNA is not the limiting factor investigating *cyp 51A* mutations from isolates.

We detected the L98H alteration without TR34 in a lung biopsy of patient 1 in 2010. The L98H mutation alone does not mediate azole resistance [12]. The L98H alteration in combination with the TR34 was detected in the brain tissue sample of patient 2 in 2011 and in the BAL sample of patient 3 in 2012. In patient 2 suffering from ALL the clinical impact of the detected mutation pattern was not assessable, because the patient was treated at the time of brain biopsy both with an azole antifungal combined with amphotericin B. The TR34/L98H alterations were also detected in the BAL sample of patient 3 and in the corresponding clinical isolate (A 4813 from the University Hospital of Cologne) of this patient [10]. The direct detection of the mutations in the clinical BAL sample of patient 3 and after that also in the corresponding clinical isolate was an important confirmation of our method detecting the *cyp51 A* mutations directly from primary clinical samples.

Investigation of 50 clinical samples for the TR46 alteration revealed no mutations after PCR and DNA sequence analysis of the PCR fragments in our series. The TR46 alteration was only detectable in the known TR46/Y121F/T289A *A. fumigatus* isolate (A12519), as a proof of the feasibility of this TR46 PCR assay.

The first documented hematological patient suffering from a multi-azole resistant IA was detected in Spain in 2003 and described in 2013 [6]. In 2007 Verweij et al., described two hematological patients with clinical isolates from 2006 with multi-azole *A. fumigatus* infections which died under azole therapy [7]. Van der Linden et al. published the investigation of *A. fumigatus* isolates collected from 2007 to 2009 and from 2009 to 2011 and described in 2011 and 2013 altogether eleven well documented hematological patients suffering from multi-azole resistant IA caused by *cyp51 A* TR34/L98H and TR46/Y212F/T289A alterations [4], [8]. Recently, Bader et. al. described 17 azole resistant clinical *A. fumigatus* isolates from a total of 527 patients investigated in Germany. From these isolates only one was originated from a hematological patient [11].

In culture based diagnosis of multi-azole resistance the amount of the pathogen is the limiting factor when investigating hematological patients because positive cultural yields are rarely achieved in this group of patients. It is highly likely that multi-azole resistance is underdiagnosed in this patients' population resulting in the clinical consequence that 9/11 (81.8%) [4], [8] and 1/1 [100%] hematological patients [11] with multi-azole resistant IA died under azole therapy, thus underlining the necessity of using non-culture based assays for both the detection of *Aspergillus* spp. and azole resistance directly from clinical samples. In our series the incidence of multi-azole resistance mediating mutations in clinical samples amounts to 1.94% (3/155 patients) in a strictly defined population of patients with hematological malignancies.

Our results demonstrate that azole resistance and *cyp51 A* mutations also occur in neutropenic, hematological patients in Germany, and that the direct detection of *cyp51 A* mutations from primary clinical samples is a huge advantage in this group of patients. Further epidemiological investigations of a higher number of clinical samples and isolates have to clarify definitively the incidence of azole resistance in hematological patients.

We consider our molecular assays of high epidemiological and clinical relevance to detect azole resistance directly from primary clinical samples of hematological patients and to allow significantly quicker targeted antifungal therapy in patients with IA.
